# Systemic Inflammatory Response Index (SIRI) is associated with all-cause mortality and cardiovascular mortality in population with chronic kidney disease: evidence from NHANES (2001–2018)

**DOI:** 10.3389/fimmu.2024.1338025

**Published:** 2024-03-15

**Authors:** Linguo Gu, Zhenkun Xia, Bei Qing, Wei Wang, Hongzuo Chen, Juan Wang, Ying Chen, Zhengling Gai, Rui Hu, Yunchang Yuan

**Affiliations:** Department of Thoracic Surgery, the Second Xiangya Hospital of Central South University, Changsha, Hunan, China

**Keywords:** SIRI, CKD, mortality, cardiovascular risk, NHANES

## Abstract

**Objective:**

To examine the correlation between SIRI and the probability of cardiovascular mortality as well as all-cause mortality in individuals with chronic kidney disease.

**Methods:**

A cohort of 3,262 participants from the US National Health and Nutrition Examination Survey (NHANES) database were included in the study. We categorized participants into five groups based on the stage of chronic kidney disease. A weighted Cox regression model was applied to assess the relationship between SIRI and mortality. Subgroup analyses, Kaplan–Meier survival curves, and ROC curves were conducted. Additionally, restricted cubic spline analysis was employed to elucidate the detailed association between SIRI and hazard ratio (HR).

**Results:**

This study included a cohort of 3,262 individuals, of whom 1,535 were male (weighted proportion: 42%), and 2,216 were aged 60 or above (weighted proportion: 59%). Following adjustments for covariates like age, sex, race, and education, elevated SIRI remained a significant independent risk factor for cardiovascular mortality (HR=2.50, 95%CI: 1.62-3.84, p<0.001) and all-cause mortality (HR=3.02, 95%CI: 2.03-4.51, p<0.001) in CKD patients. The restricted cubic spline analysis indicated a nonlinear relationship between SIRI and cardiovascular mortality, with SIRI>1.2 identified as an independent risk factor for cardiovascular mortality in CKD patients.

**Conclusion:**

Heightened SIRI independently poses a risk for both all-cause and cardiovascular mortality in chronic kidney disease patients, with potentially heightened significance in the early stages (Stage I to Stage III) of chronic kidney disease.

## 1 Introduction

Chronic kidney disease (CKD), characterized by a low estimated glomerular filtration rate (eGFR) or elevated albuminuria, is identified as one of the top 10 contributors to poor prognosis globally (1). Epidemiology shows that approximately 15% -20% of people worldwide suffer from chronic kidney disease, and adverse outcomes caused by chronic kidney disease are an issue that cannot be ignored (2, 3). Among all the adverse outcomes caused by CKD, cardiovascular disease (CVD), as a leading cause of death, is one of the most important and severe (2, 4, 5). Patients with CKD typically present with hypertension, atherosclerosis, lipid metabolism abnormalities, mineral and bone disorders, chronic inflammation, and oxidative stress (6–12). These alterations commonly result from a decline in the glomerular filtration rate (9). Notably, chronic inflammation and oxidative stress can exacerbate endothelial damage, leading to the formation of unstable atherosclerotic plaques (6, 13–15). The intricate and complex mechanisms contributing to the onset of chronic inflammation and oxidative stress encompass the accumulation of metabolic byproducts, injury to renal tubular and glomerular cells, activation of the immune system, and disruption of endocrine regulation, among other factors (16–18). Under the collective influence of these factors, patients with CKD are more susceptible to cardiovascular events. Recent investigations reveal an upregulation in the surface expression of IL-1α on monocytes in individuals afflicted with acute myocardial infarction (AMI) and CKD. IL-1α, identified as a pivotal regulatory factor in the inflammatory processes associated with myocardial infarction and CKD, demonstrates a significant correlation with the incidence of cardiovascular events (19). Bioinformatics analysis reveals that the PI3K-AKT signaling pathway and ICAM1-mediated neutrophil infiltration are potential common pathogenic mechanisms in CKD (20). Therefore, monocytes and neutrophils play pivotal roles in the pathogenesis of CKD.

As stated below, existing research indicates that certain inflammatory markers are considered independent risk factors for mortality. In cardiovascular diseases, inflammatory markers have been employed to assess all-cause mortality in CVD (21–24) and acute coronary syndrome (ACS) (25–27). Evidence has emerged linking two-line inflammation indices, such as the neutrophil-to-lymphocyte ratio (NLR), platelet-to-lymphocyte ratio (PLR), and monocyte-to-lymphocyte ratio (MLR), to cardiovascular mortality (27–29). Systemic Inflammatory Response Index (SIRI), a novel composite index, integrates three independent white blood cell subsets (30). SIRI was initially used for the assessment of tumor prognosis (31, 32), however, the latest findings suggest that an elevated SIRI strongly correlates with unfavorable outcomes in acute coronary syndrome, cardiovascular event mortality, arrhythmias among stroke patients, and major adverse cardiovascular events following percutaneous coronary intervention (PCI) (33–35). A limited number of studies suggest that SIRI may offer distinct predictive utility in the prediction of acute kidney injury resulting from abdominal trauma (36). An analysis of SIRI elevation and cardiovascular mortality risk in hypertensive patients revealed that those in the fourth quartile of SIRI had a 2.9-fold increased risk of cardiovascular death compared to patients in the first quartile (37). Similar conclusions were confirmed in a follow-up cohort study involving 42,875 participants in the United States (30). In a cohort study conducted after peritoneal dialysis, it was observed that patients in the high SIRI level group had a cardiovascular mortality risk of approximately 2.8 times higher than that of the low-level group (38). These studies strongly suggest that an elevation in SIRI levels may be an independent risk factor for cardiovascular mortality. However, the relationship between SIRI and the risk of cardiovascular mortality in patients with CKD is unclear. Nonetheless, there is a paucity of relevant studies regarding whether SIRI can predict the risk of cardiovascular mortality in individuals afflicted by CKD.

Therefore, in order to explore the potential relationship between SIRI levels and the risk of cardiovascular and all-cause mortality in CKD individuals, we conducted a retrospective analysis using data from participants in the National Health and Nutrition Examination Survey (NHANES). This study serves as a foundational step for future research in this field.

## 2 Materials and methods

### 2.1 Data and study participants

The data utilized in this study were obtained from the National Health and Nutrition Examination Survey (NHANES, https://www.cdc.gov/nchs/nhanes/). NHANES database is a publicly accessible resource under the guidance of the National Center for Health Statistics (NCHS), which is responsible for data collection and quality control. Data extraction from the NHANES database was carried out using a rigorous stratified, multistage, and complex sampling methodology. This robust approach facilitated the acquisition of essential demographic particulars, physical assessments, biochemical blood analysis, as well as comprehensive responses to questionnaires about dietary patterns and medical histories. These data collectively provided an extensive portrayal of the health status and lifestyle factors prevalent within the U.S. population. This survey adheres to ethical guidelines, with all participants having provided informed consent before their inclusion.

**Figure 1:**
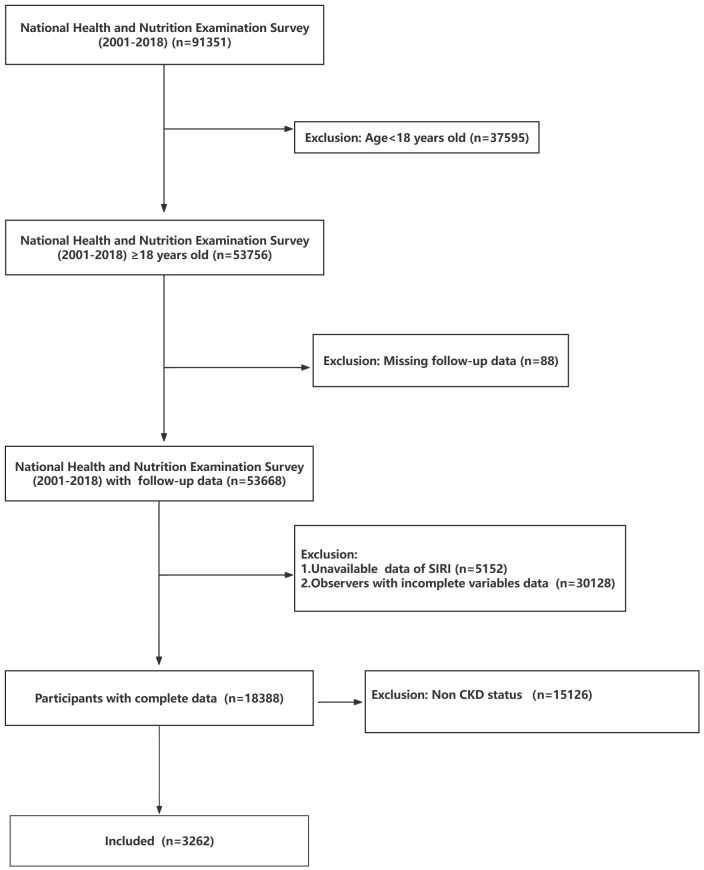
Flowchart of the participant’s selection from NHANES 2021-2018. In the NHANES database, each participant is given a unique SEQN identifier. Across various data collection periods, data related to the same participant is consolidated using the distinct SEQN identifier. CKD, Chronic kidney disease.

Our study collected data from 2001 to 2018, encompassing a total of 91,351 participants. Initially, individuals under 18 years of age (n=37,595) and those without follow-up data (n=88) were excluded. Subsequently, a total of 5,152 individuals were excluded from the analysis due to insufficient information on complete blood cell counts, preventing the calculation of SIRI. Next, those with incomplete covariate information (n=30,128) were removed. Finally, we banned 15,126 participants who were not in CKD status. Eventually, 3262 participants were included in this study (**Figure 1**).

### 2.2 Exposure variable

Neutrophil, lymphocyte and monocyte counts were measured using automated hematology analyzing devices. SIRI is calculated by the following formula (39, 40):


SIRI= (monocyte count×neutrophils count)/lymphocytes count

### 2.3 Covariates

The demographic data encompassed several variables, including age(categorized into 18-39, 40–59, ≥ 60 years old, referred to literature (41)), gender, race, education level, the ratio of family income to poverty (PIR), smoking and drinking habits, dialysis, systolic blood pressure (SBP), and diastolic blood pressure (DBP). Serum albumin, serum creatinine, alanine transaminase (ALT), aspartate transaminase(AST), triglycerides (TG), glycohemoglobin(GHB) and fasting blood glucose were performed under a unified standard. The laboratory utilizes the Jaffe rate method (kinetic alkaline picrate) to determine the concentration of creatinine in serum and plasma. A solid-phase fluorescent immunoassay (FIA) was employed for the quantification of human urinary albumin, while the enzymatic method was utilized to measure urinary creatinine levels. Urinary albumin and urinary creatinine are measured in random urine collected. To calculate the urinary albumin to creatinine ratio (ACR) and estimated glomerular filtration rate (eGFR), we employed the following formulas (41, 42):


ACR (mg/g) =urinary albumin(mg/dL)/urine creatinine(g/dL)

$$\text{eGFR }\left(\text{ml}/\text{min}/1.73{\text{m}}^{2}\right)=1419{\left(\frac{Scr}{\kappa },1\right)}^{\alpha }\times \text{max}{\left(\frac{Scr}{\kappa },1\right)}^{-1.209}\times 0.993^{\text{Age}}\times 1.018\left(\text{if female}\right)\times 1.159(\text{if black)}$$

It is important to note that Scr denotes serum creatinine concentration (mg/dL), and κ is 0.9 for males and 0.7 for females, and α is −0.411 for males and −0.329 for females.

We defined hypertension as meeting any of the following criteria: (a) self-report of a doctor’s diagnosis of hypertension; (b) current prescription for hypertension medication; (c) three consecutive measurements of systolic blood pressure (SBP) > 140 mmHg and/or diastolic blood pressure (DBP) > 90 mmHg on different days. For diabetes, the definition included meeting any of the following conditions (43): (a) a prior diagnosis of diabetes by a healthcare professional or current use of diabetes medications; (b) fasting blood glucose (FBG) ≥ 7.0 mmol/L; (c) glycated hemoglobin (HbA1C) ≥ 6.5%. According to a comprehensive review of the literature, the assessment of CKD status is based on the following criteria (5, 41, 44–46): (a) eGFR< 60 ml/min/1.73 m^2^, (b) ACR > 30 mg/g. The CKD stage is based on the calculated results of eGFR (ml/min/1.73m^2^). The classification of CKD stage and the albuminuria grade are by international guidelines (44, 47) (CKD Stage I: eGFR≥90, Stage II: 60≤eGFR<90, Stage III:30≤eGFR<60, Stage IV: 15≤eGFR<30, Stage V: eGFR<15. Albuminuria grade: Mildly:<30mg/g, Moderately:30-300mg/g, Severely:>300mg/g).

### 2.4 Outcome assessment

The vital status of participants was verified by cross-referencing with the National Death Index (NDI) (https://ftp.cdc.gov/pub/Health_Statistics/NCHS/datalinkage/linked_mortality/).

All-cause mortality encompassed deaths from any cause. Cardiovascular mortality was defined as I00–I09, I11, I13, I20–I51, or I60–I69, as recorded in the National Death Index (48). This definition encompassed both cardiovascular disease (CVD) and cerebrovascular disease. For brevity, we may refer to cardiovascular mortality as CVD mortality. In the codebook for the National Center for Health Statistics (NCHS) 2019 public-use linked mortality files (**Supplementary File 1**), the ucode_leading for CVD mortality is represented as 001 and 005.

### 2.5 Statistical analysis

We performed data analysis using the R project (version 4.1.1). Given the complex, multi-stage, and multi-stratified sampling approach used in the NHANES database, we incorporated weighting variables as guided by the data provider’s usage instructions. The “gtsummary” package in the R project was employed for weighted statistical analysis. We initiated our analysis of continuous variables with a normal distribution test, which indicated a departure from a normal distribution. Consequently, we represented these non-normally distributed data by reporting the median, along with the 25% and 75% percentiles, and applied the Wilcoxon test for statistical analysis. As for categorical variables, we illustrated them through numerical values and proportions and conducted statistical analysis employing the chi-square test.

In order to mitigate potential statistical biases, we adopted a weighted Cox regression model rather than the traditional regression model for our survival analysis of the study population. We employed Hazard Ratios (HR) accompanied by 95% Confidence Intervals (CI) to evaluate the impact of SIRI on survival. Next, we conducted a proportional hazards assumption test for the Cox regression models, affirming that our models adhered to the assumption of proportional hazards. Subsequently, we established three Cox regression models with variable adjustments to assess the association between SIRI and survival. Following this, subgroup analyses were conducted. We performed weighted Cox regression analyses on the association between SIRI and the risk of mortality across seven subgroups, including age, gender, the presence of hypertension or diabetes, dialysis status, distinct CKD stages, and diverse albuminuria grades. After that, we generated Kaplan-Meier (KM) survival curves to compare the survival status among different SIRI groups. To elucidate the potential interactions between these seven subgroups and the risk of mortality, we conducted interaction analyses concurrently.

We constructed receiver operating characteristic (ROC) curves to evaluate the specificity and sensitivity of SIRI in predicting mortality. Restricted cubic spline analysis, a scientific and commonly utilized analytical method, is employed to investigate the potential nonlinear relationship between two variables (49, 50). This method enables curve fitting through restricted cubic splines. In the context of our Cox regression model, restricted cubic spline analysis is used to elucidate how the hazard ratio (HR) values vary with changes in SIRI. Statistical significance was considered at a p-value< 0.05 in all hypothesis tests.

## 3 Results

### 3.1 Population characteristics

This study encompassed a cohort of 3,262 individuals. Among them, 1,535 were male (weighted proportion: 42%), and 2,216 were aged 60 or above (weighted proportion: 59%). According to the CKD staging, these individuals are classified into five groups. The populations in different groups exhibit statistical differences in terms of age, racial distribution, family income situation, drinking habits, BMI, fasting blood glucose, lipid profiles, liver function indicators, and serum creatinine levels. However, no statistically significant differences were found in terms of gender distribution, smoking habits, and educational levels (**Table 1**).

**Table 1:** Demographic and clinic characteristics.

CKD Stage								
Characteristic	N^1^	Overall, N = 3262 (100%)^2^	Stage I, N = 948 (33%)^2^	Stage II, N = 766 (21%)^2^	Stage III, N = 1401 (42%)^2^	Stage IV, N = 109 (2.8%)^2^	Stage V, N = 38 (0.9%)^2^	P Value^3^
**Age (Year)**	3,262							**<0.001**
18-39		367 (15%)	323 (41%)	24 (5%)	13 (1%)	5 (6%)	2 (7%)	
40-59		679 (26%)	401 (44%)	167 (28%)	95 (10%)	7 (8%)	9 (28%)	
≥ 60		2,216 (59%)	224 (14%)	575 (67%)	1,293 (89%)	97 (86%)	27 (65%)	
**Gender**	3,262							0.066
Female		1,727 (58%)	548 (59%)	364 (53%)	736 (59%)	63 (66%)	16 (42%)	
Male		1,535 (42%)	400 (41%)	402 (47%)	665 (41%)	46 (34%)	22 (58%)	
**Race**	3,262							**<0.001**
Mexican American		491 (8%)	247 (15%)	126 (6%)	102 (2%)	8 (5%)	8 (13%)	
Other Hispanic		210 (4%)	79 (6%)	53 (5%)	70 (2%)	7 (4%)	1 (3%)	
Non- Hispanic White		1,676 (70%)	297 (56%)	365 (69%)	938 (83%)	64 (68%)	12 (48%)	
Non- Hispanic Black		655 (12%)	243 (16%)	154 (12%)	218 (8%)	26 (18%)	14 (29%)	
Other Race		230 (6%)	82 (7%)	68 (8%)	73 (4%)	4 (4%)	3 (6%)	
**PIR**	3,262	2.33 (1.31, 4.13)	2.15 (1.16, 3.99)	2.23 (1.26, 4.10)	2.51 (1.49, 4.30)	1.93 (1.32, 3.49)	1.91 (0.85, 2.72)	**0.001**
**Education**	3,262							0.3
<High School		1,058 (23%)	311 (23%)	273 (24%)	419 (21%)	42 (34%)	13 (24%)	
High School or some College		1,672 (57%)	485 (57%)	376 (56%)	735 (59%)	55 (56%)	21 (66%)	
College or Above		532 (20%)	152 (21%)	117 (19%)	247 (20%)	12 (11%)	4 (10%)	
**Smoking**	3,262							0.6
No		1,642 (51%)	497 (52%)	361 (48%)	713 (51%)	52 (54%)	19 (62%)	
Yes		1,620 (49%)	451 (48%)	405 (52%)	688 (49%)	57 (46%)	19 (38%)	
**Drinking**	3,262							**0.003**
No		940 (27%)	254 (24%)	205 (23%)	431 (30%)	34 (37%)	16 (50%)	
Yes		2,322 (73%)	694 (76%)	561 (77%)	970 (70%)	75 (63%)	22 (50%)	
**Hypertension**	3,262							**<0.001**
No		931 (33%)	443 (53%)	187 (30%)	281 (20%)	16 (13%)	4 (7%)	
Yes		2,331 (67%)	505 (47%)	579 (70%)	1,120 (80%)	93 (87%)	34 (93%)	
**Diabetes**	3,262							**0.011**
No		2,005 (66%)	599 (70%)	438 (61%)	889 (67%)	58 (59%)	21 (51%)	
Yes		1,257 (34%)	349 (30%)	328 (39%)	512 (33%)	51 (41%)	17 (49%)	
**Dialysis**	3,262							**<0.001**
No		3,231 (99%)	948 (100%)	765 (100%)	1,397 (100%)	104 (96%)	17 (34%)	
Yes		31 (1%)	0 (0%)	1 (0%)	4 (0%)	5 (4%)	21 (66%)	
**BMI (kg/m^2^)**	3,262	28.69 (24.80, 34.00)	29.26 (24.00, 35.10)	28.90 (25.30, 34.93)	28.26 (25.07, 32.40)	28.80 (24.98, 33.44)	24.26 (22.12, 27.49)	**0.006**
**Creatinine (mg/dL)**	3,262	1.00 (0.78, 1.23)	0.70 (0.64, 0.81)	0.92 (0.80, 1.06)	1.22 (1.06, 1.40)	2.20 (1.94, 2.50)	6.30 (4.34, 7.44)	**<0.001**
**ALT(U/L)**	3,262	20.00 (15.00, 26.00)	21.00 (16.00, 32.00)	20.00 (16.00, 27.00)	19.00 (15.00, 24.00)	16.00 (12.00, 19.79)	16.00 (13.00, 22.07)	**<0.001**
**AST(U/L)**	3,262	23.00 (19.00, 28.00)	22.00 (19.00, 28.00)	24.00 (20.00, 28.00)	23.00 (20.00, 27.00)	21.37 (18.00, 26.00)	18.00 (16.00, 22.84)	**0.003**
**Triglycerides (mmol/L)**	3,262	1.32 (0.91, 1.98)	1.29 (0.85, 2.00)	1.33 (0.95, 2.15)	1.33 (0.95, 1.91)	1.60 (1.08, 2.26)	1.24 (0.89, 1.66)	**0.048**
**Glycosylated hemoglobin (%)**	3,262	5.70 (5.40, 6.20)	5.57 (5.20, 6.20)	5.70 (5.40, 6.50)	5.80 (5.50, 6.20)	5.90 (5.50, 6.47)	5.70 (5.30, 6.63)	**<0.001**
**Fasting blood glucose (mmol/L)**	3,262	5.83 (5.29, 6.94)	5.71 (5.11, 6.97)	6.05 (5.50, 7.33)	5.83 (5.38, 6.77)	6.05 (5.31, 7.05)	5.05 (4.45, 6.25)	**<0.001**
**Hemoglobin (g/dL)**	3,262	14.00 (12.90, 15.10)	14.40 (13.30, 15.50)	14.20 (13.30, 15.40)	13.80 (12.70, 14.80)	12.30 (11.00, 13.28)	11.30 (9.82, 12.81)	**<0.001**
**eGFR(ml/min/1.73m^2^)**	3,262	67.71 (52.76, 98.75)	107.03 (98.75, 117.63)	76.87 (68.45, 83.58)	52.42 (45.62, 56.77)	24.14 (20.64, 26.94)	8.35 (6.94, 11.95)	**<0.001**
**Albuminuria Grade**	3,262							**<0.001**
Mildly		1,006 (32%)	0 (0%)	0 (0%)	969 (75%)	33 (31%)	4 (9%)	
Moderately		1,867 (58%)	839 (90%)	651 (87%)	328 (19%)	38 (32%)	11 (35%)	
Severely		389 (10%)	109 (10%)	115 (13%)	104 (6%)	38 (37%)	23 (57%)	
**ACR (mg/g)**	3,262	41.83 (13.90, 90.37)	57.74 (39.61, 115.52)	60.72 (39.80, 125.83)	11.04 (5.82, 30.45)	69.75 (19.65, 581.58)	365.29 (113.99, 2,374.97)	**<0.001**
**SIRI**	3,262	1.20 (0.80, 1.84)	1.02 (0.67, 1.59)	1.28 (0.86, 2.02)	1.27 (0.88, 1.90)	1.52 (1.03, 2.42)	1.50 (0.95, 1.90)	**<0.001**

^1^N not Missing (unweighted).

^2^median (IQR) for continuous; n (%) for categorical.

^3^chi-squared test with Rao & Scott's second-order correction; Wilcoxon rank-sum test for complex survey samples.

The bold values denote statistically significant difference at p < 0.05 level.

Advanced stages of CKD appear to be more prevalent in older individuals, those from economically disadvantaged households, individuals with comorbid hypertension, those undergoing dialysis, and those with elevated levels of serum creatinine, ACR, and SIRI. Participants with lower SIRI scores tend to exhibit characteristics similar to individuals with higher glomerular filtration rates (eGFR) and hemoglobin levels.

### 3.2 SIRI is an independent predictor of mortality

Three models were developed to assess the independent functionality of SIRI and its correlation with mortality. The investigation entailed the utilization of multivariate weighted Cox regression to examine the interconnection between SIRI and mortality, with meticulous adjustments made to account for potential confounding variables. The findings, inclusive of Hazard Ratios (HRs) and their corresponding 95% CIs, have been presented for comprehensive analysis (**Table 2**). It is important to note that in the weighted Cox regression analysis, all participants will be reclassified into four groups based on the quartiles of SIRI (Q1:<0.661, 0.661≤Q2<0.978, 0.978≤Q3<1.460, Q4:≥1.460).

**Table 2:** Multivariate Cox regression analysis of SIRI and mortality.

	Characteristic	HR^1^	95% CI^1^	p-value	P for trend	Characteristic	HR^1^	95% CI^1^	p-value	P for trend
	SIRI for CVD mortality					SIRI for All-cause mortality				
**Model1**	Q1	Reference	—		**p<0.001**	Q1	Reference	—		**p<0.001**
	Q2	1.06	0.58, 1.93	0.849		Q2	1.61	1.00, 2.58	**0.048**	
	Q3	1.56	0.95, 2.57	0.082		Q3	1.97	1.26, 3.07	**0.003**	
	Q4	2.50	1.62, 3.84	**<0.001**		Q4	3.02	2.03, 4.51	**<0.001**	
**Model2**	Q1	Reference	—		**p<0.001**	Q1	Reference	—		**p<0.001**
	Q2	0.83	0.45, 1.50	0.533		Q2	1.29	0.83, 2.02	0.257	
	Q3	1.20	0.75, 1.93	0.453		Q3	1.59	1.05, 2.41	**0.030**	
	Q4	1.72	1.10, 2.68	**0.017**		Q4	2.19	1.49, 3.23	**<0.001**	
**Model3**	Q1	Reference	—		**p<0.001**	Q1	Reference	—		**p<0.001**
	Q2	0.81	0.46, 1.44	0.481		Q2	1.21	0.78, 1.88	0.397	
	Q3	1.16	0.73, 1.85	0.531		Q3	1.50	0.99, 2.28	0.057	
	Q4	1.74	1.13, 2.67	**0.012**		Q4	2.05	1.39, 3.04	**<0.001**	

1HR = Hazard Ratio, CI = Confidence Interval.

Model 1: No confounding factors were adjusted.

Model2 :Adjusted for Age、Gender、 Race、Education level、PIR、Dialysis.

Model3:Adjusted for Age、Gender、 Race、Educationlevel、BMI、Hypertension、Dialysis、Diabetes、Smoking、Drinking、Creatinine、ALT、AST、 ACR、Triglycerides、 Glycosylated hemoglobin 、 Fasting blood glucose.

The bold values denote statistically significant difference at p < 0.05 level.

In Model 1, no covariates were adjusted. In Model 2, we made adjustments for age, gender, race, education level, poverty status (PIR), and dialysis status. In Model 3, we further adjusted for BMI, hypertension, diabetes, alcohol consumption, smoking status, serum creatinine, fasting blood glucose, glycated hemoglobin, lipid levels, ACR, and transaminase levels. The Cox regression results indicate that higher SIRI levels are associated with an increased risk of cardiovascular mortality (Q4 vs. Q1). Compared to participants in the first quartile of SIRI, those in the fourth quartile of SIRI experienced a 2.5-fold increase in cardiovascular mortality risk and a 3.02-fold increase in all-cause mortality risk.

**Figure 2:**
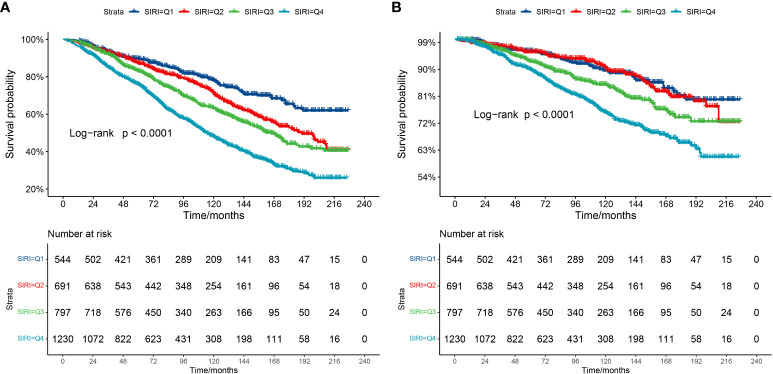
Kaplan-Meier survival curve. **(A)** Kaplan-Meier survival curve for all-cause mortality. **(B)** Kaplan-Meier survival curve for cardiovascular mortality. In the Kaplan-Meier curves, the population is stratified into four groups (Q1, Q2, Q3, Q4) based on the quartiles of SIRI, and statistical analysis is conducted using the log-rank test.

The Kaplan–Meier curve (**Figure 2**) corroborates the aforementioned conclusion. It demonstrates that individuals with higher SIRI scores experience a more significant decline in survival over a 20-year follow-up when compared to those with lower SIRI levels in CKD populations (log-rank P for trend<0.0001).

### 3.3 Subgroup analysis and interaction analysis

To determine the consistency of the relationship between SIRI and mortality across various subgroups of the population, subgroup analyses were conducted within specific categories, including age, gender, the presence of hypertension and diabetes, dialysis status, CKD stage, and albuminuria grade.

**Figure 3:**
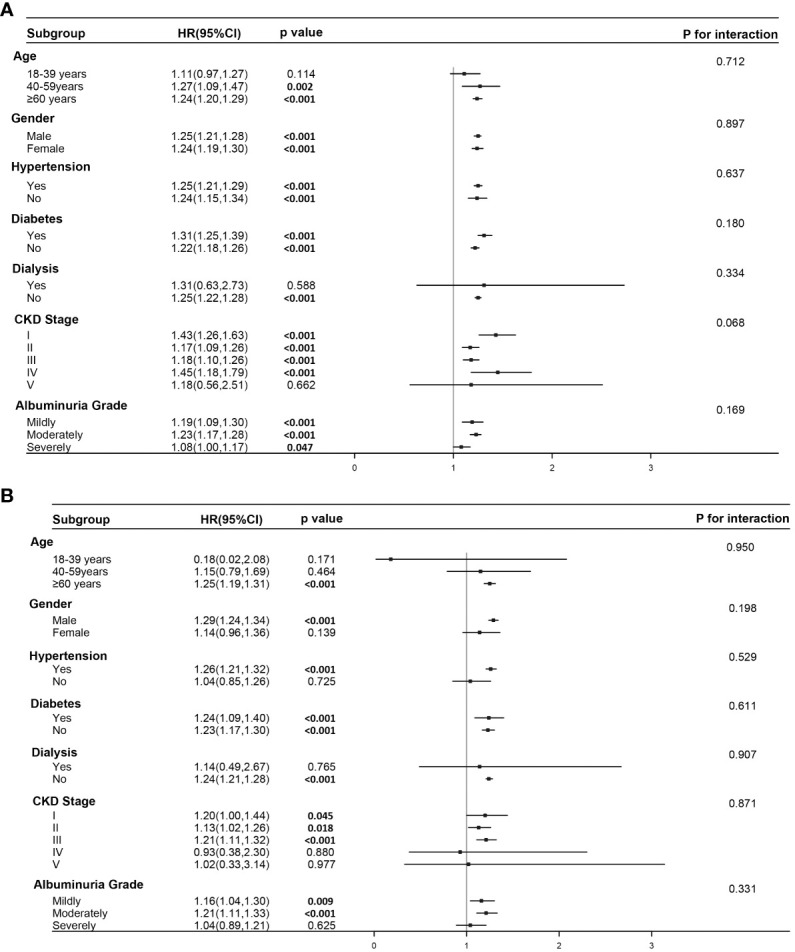
Subgroup analysis and interaction analysis. **(A)** Subgroup analysis for the association between SIRI and all-cause mortality. **(B)** Subgroup analysis for the association between SIRI and cardiovascular mortality. Subgroup analysis were conducted using a weighted Cox regression model. In the forest plot, a weighted Cox regression model is still employed. Interaction analysis utilizes the Likelihood Ratio Test.

The results from the forest plot (**Figure 3**) reveal that elevated SIRI remains an independent risk factor for all-cause mortality in individuals aged over 40, not undergoing dialysis, and with CKD stages I-IV. This association holds regardless of the presence of comorbid hypertension and diabetes or the severity of urinary albumin levels. However, the conclusion that elevated SIRI increases the risk of cardiovascular mortality appears to be established only in male participants aged over 60, with comorbid hypertension but not undergoing dialysis. Furthermore, among individuals in CKD stages, I-III, or those with mild to moderate albuminuria, elevated SIRI is associated with an increased risk of cardiovascular mortality. Interaction analysis indicates the absence of interaction between SIRI and these seven factors in jointly influencing cardiovascular or all-cause mortality. This implies that SIRI independently serves as a factor, contributing to an increased risk for both types of mortality.

### 3.4 Sensitivity and specificity analysis

**Figure 4:**
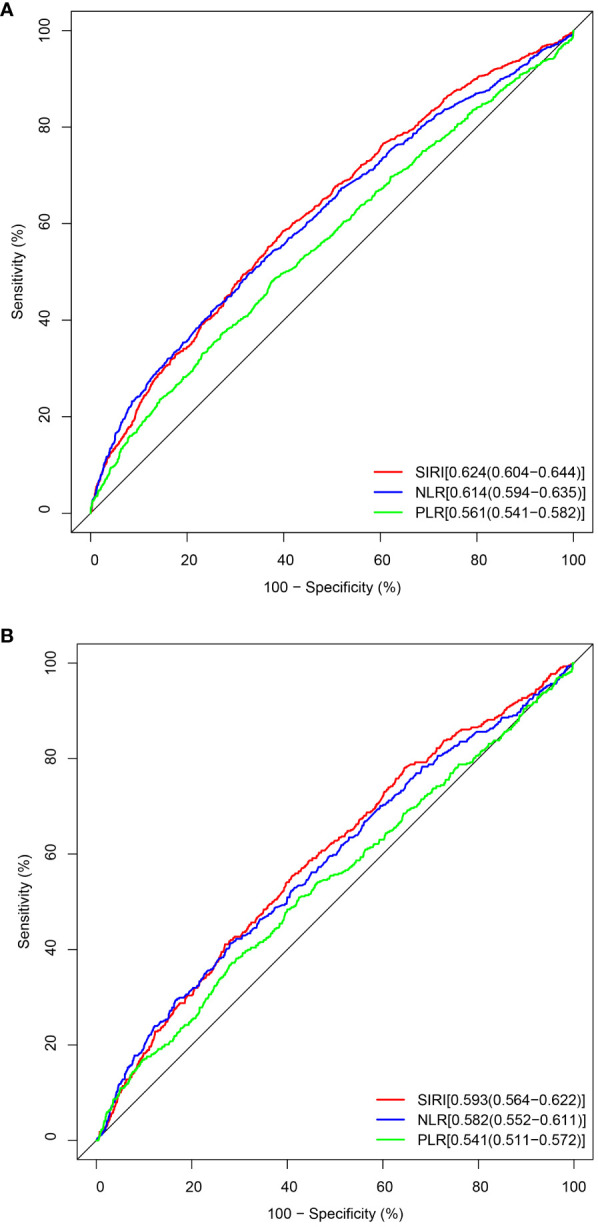
The ROC value of SIRI, NLR, and PLR in predicting outcomes in CKD patients. **(A)** ROC curve analysis of SIRI and all-cause mortality. The AUC of SIRI was 0.624 with a sensitivity of 0.585 and a specificity of 0.601. **(B)** ROC curve analysis of SIRI and cardiovascular mortality. The AUC of SIRI was 0.593 with a sensitivity of 0.555 and a specificity of 0.589.

The establishment of the Receiver Operating Characteristic curve (ROC, **Figure 4**) is integral for evaluating the sensitivity and specificity of SIRI as a prognostic diagnostic tool. SIRI’s predictive capacity for all-cause mortality is exemplified by an Area Under the Curve (AUC) of 0.624 (95% CI: 0.604, 0.644). Likewise, the predictive performance of SIRI for cardiovascular mortality yields an AUC of 0.593 (95% CI: 0.564, 0.622).

### 3.5 Restricted cubic spline regression analysis

**Figure 5:**
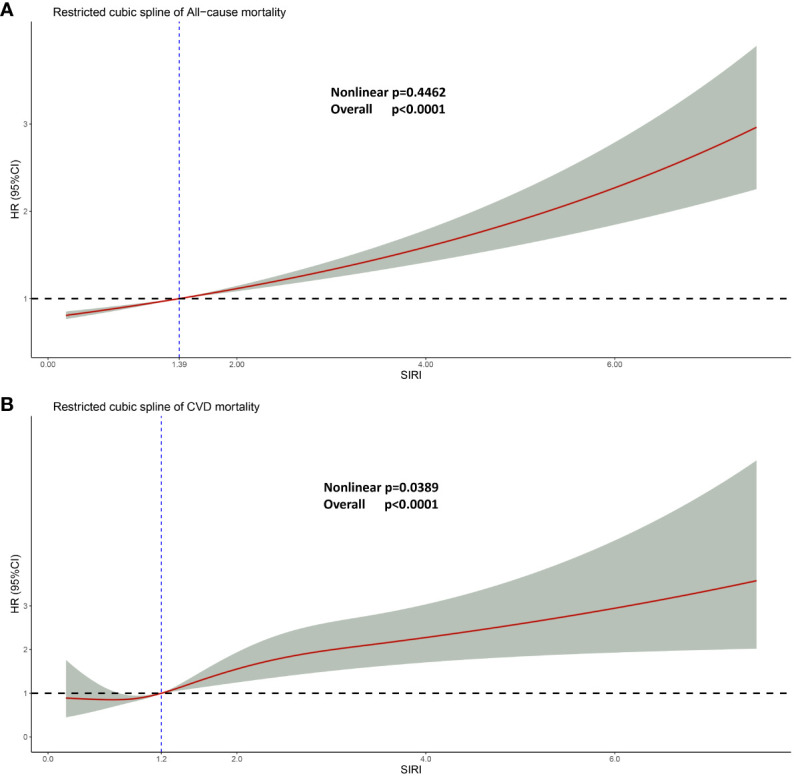
Restricted cubic spline regression analysis. **(A)** Linear relationship between SIRI and all-cause mortality. **(B)** Non-linear relationship between SIRI and cardiovascular mortality. For both **(A, B)**, adjusted for age, gender, race, education, smoking, drinking, hypertension, diabetes, ALT, AST, fasting blood glucose, serum creatinine, total cholesterol, and glycosylated hemoglobin. Likelihood Ratio Test was used in the Restricted cubic spline regression analysis analysis.

In order to elucidate the specific relationship between SIRI and HR (Hazard Ratio), the restricted cubic spline regression analysis was conducted (**Figure 5**). In the examination of the relationship between SIRI and the Hazard Ratio (HR) for cardiovascular mortality, we discovered a non-linear correlation (Nonlinear p-value = 0.0389, which is<0.05, indicating a significant nonlinear relationship between SIRI and HR for CVD). Therefore, we utilized a non-linear model to elucidate their association. The SIRI value corresponding to an HR of 1 is 1.2. When SIRI is<1.2, there is a decrease in cardiovascular mortality risk within a certain range as SIRI increases. However, when SIRI > 1.2, there is an increase in cardiovascular mortality risk, and as SIRI continues to increase, the HR for cardiovascular mortality changes gradually and levels off.

Interestingly, in the context of all-cause mortality, SIRI and HR exhibit a linear association (non-linear p = 0.4462). As SIRI increases, HR follows suit, and this trend persists beyond the threshold of 1.39, at which point HR surpasses 1.0.

## 4 Discussion

This is a multicenter, large-scale, and complex sampling cross-sectional study that investigates the relationship between SIRI levels and cardiovascular mortality as well as all-cause mortality in 3262 interviewed individuals with chronic kidney disease in the United States. To the best of our knowledge, this is the first report to explore the association between these two factors. Using a weighted Cox regression model, we have identified a linear relationship between SIRI and all-cause mortality in patients with chronic kidney disease, while a non-linear relationship was observed with cardiovascular mortality (**Figure 5**). Notably, SIRI has emerged as an independent risk factor for both all-cause mortality and cardiovascular mortality in patients with chronic kidney disease. These findings strongly support the notion that SIRI plays a significant role in predicting mortality outcomes in this patient population.

Inflammation is regarded as a fundamental component in the pathophysiological processes associated with chronic kidney disease (51). Inflammation additionally triggers abnormal activation of neutrophils and the monocyte-macrophage system, subsequently inducing the release of inflammatory factors. These factors contribute to vascular endothelial damage and platelet aggregation (52–55). Among these factors, the differentiation of monocytes into macrophages plays a crucial role in the pathogenesis of ischemic heart disease (56). Pro-inflammatory cytokines such as TNF, IL-1β, and IL-6, secreted by monocytes, enhance their migratory potential towards tissue sites of inflammation and are associated with an increased risk of cardiovascular disease in patients with CKD (57). A study of a mouse myocardial infarction model reveals that after myocardial infarction, despite the predominance of N1 neutrophils, the percentage of N2 neutrophils increased post-MI from 2.4 ± 0.6% on Day 1 to 18.1 ± 3.0% on Day 7 (58). This implies that within the first week after a heart attack, the total number of neutrophils increases. A cross-sectional study revealed a significant reduction in the total number of lymphocytes in the peripheral blood of patients with end-stage renal disease compared to the healthy population (59). In essence, the increased counts of monocytes and neutrophils, coupled with reduced lymphocyte numbers, may offer a plausible explanation for the heightened risk of all-cause and cardiovascular mortality associated with elevated SIRI levels in patients with CKD. Numerous studies have illustrated immune system dysregulation, heightened inflammatory signaling, diminished release of anti-inflammatory factors, and persistent accumulation of chronic inflammation in individuals with chronic kidney disease (60–63). Therefore, anti-inflammatory therapy is also recommended for patients in CKD stages I to III (64). Interestingly, inflammatory activities may be more pronounced during CKD stages IV and V than during stages I to III (65), which is consistent with our findings(advanced CKD stage with a higher level of SIRI). However, in patients with advanced stages of chronic kidney disease (IV-V), the primary causes of cardiovascular death are sudden cardiac death and myocardial infarction rather than inflammatory reactions (66, 67). This might be a reason that in our subgroup analysis, an elevation in SIRI appears to be unrelated to an increased risk of cardiovascular death in participants in CKD stages IV and V.

Several factors can impact the mortality of individuals with CKD, including age, gender, race, hypertension, diabetes, and lifestyle habits like smoking and drinking (68). To mitigate the influence of these variables, three Cox models were employed to elucidate the association between elevated SIRI and mortality risk while adjusting for different factors. Even after accounting for confounding variables, increased SIRI persisted as an independent risk factor for both cardiovascular and all-cause mortality in chronic kidney disease patients. Subgroup analyses further revealed that elevated SIRI retained its independent association with all-cause mortality in various CKD subgroups. The simplicity of the SIRI definition and the accessibility of data through laboratory analysis of a patient’s complete blood count enhance its applicability as a novel biomarker, offering broad prospects and significant economic benefits in clinical settings. C-reactive protein (CRP) is a commonly employed marker for inflammation detection. However, our analysis of the NHANES database revealed that CRP levels were not assessed for every participant every year (the indicator was not measured from 2011 to 2014). This suggests that a higher prevalence of missing values could introduce notable statistical analysis errors. Consequently, we opted not to incorporate this variable into our study. Additionally, it’s worth noting that the cost associated with CRP measurement is higher compared to that of a complete blood count assessment. While SIRI exhibits a slightly superior predictive ability compared to NLR and PLR in the ROC curve, the overall predictive performance of SIRI is still constrained (AUC approximately 0.6). This result is consistent with the AUC reported in previous literature (37). We speculate that this may be related to the relatively small number of individuals experiencing cardiovascular death events during the follow-up period. In pursuit of practicality, we conducted a restricted cubic spline analysis (**Figure 5**) to elucidate the relationship between SIRI and hazard ratio (HR). Our findings reveal a non-linear association between SIRI and the risk of cardiovascular mortality, indicating that SIRI levels exceeding 1.2 are linked to an elevated risk of cardiovascular death in individuals with chronic kidney disease. This underscores the importance of dynamic monitoring of SIRI levels during the treatment of CKD patients to mitigate the risk of cardiovascular mortality.

Our study possesses several notable strengths. Firstly, we assessed a substantial sample size of 3,262 participants, each with comprehensive clinical information and survival data. The data collection process was meticulously designed and executed by the NCHS team in the United States, ensuring a high level of reliability following rigorous quality testing. Secondly, our utilization of a weighted Cox regression model enhances the generalizability of our findings. Despite the seemingly modest sample size, the participants effectively represented diverse social strata in the United States from 2001 to 2018, rendering our analysis results widely applicable and representative. Thirdly, we identified a specific threshold for controlling SIRI levels to mitigate the risk of cardiovascular and all-cause mortality in individuals with CKD. Nevertheless, our study has certain limitations. Firstly, its cross-sectional nature means that dynamic follow-up data are lacking. Additionally, the availability of survival data from the NCHS is limited to 2018 and earlier, precluding access to more recent follow-up information. Finally, the relatively low percentage of cardiovascular deaths among the 3,262 participants introduces some statistical error, necessitating larger cohort studies for more robust conclusions in the future.

## 5 Conclusion

In summary, our study indicates that in patients with chronic kidney disease, increased SIRI level is an independent risk factor for cardiovascular mortality(SIRI>1.20) and all-cause mortality(SIRI>1.39), with potentially heightened significance in the early stages (Stage I to Stage III) of chronic kidney disease.

## Data availability statement

The original contributions presented in the study are included in the article/**Supplementary Material**. Further inquiries can be directed to the corresponding author.

## Author contributions

LG: Writing – original draft. ZX: Data curation, Writing – review & editing. BQ: Data curation, Writing – review & editing. WW: Data curation, Writing – review & editing. HC: Data curation, Writing – review & editing. JW: Methodology, Writing – review & editing. YC: Methodology, Writing – review & editing. ZG: Methodology, Writing – review & editing. RH: Methodology, Writing – review & editing. YY: Writing – review & editing.
